# Exploratory evaluation of miR-146, inflammatory biomarkers, and subjective cognitive symptoms in chronic migraine

**DOI:** 10.55730/1300-0144.6378

**Published:** 2026-06-07

**Authors:** Hale GÖK DAĞIDIR, Duygu BANDIRMALI, Merve Hilal CEREN AKGÖR, Doğa VURALLI, Meltem YALINAY, Güvem GÜMÜŞ AKAY, Hayrunnisa BOLAY

**Affiliations:** 1 Department of Medical Biochemistry, Faculty of Medicine, Yüksek İhtisas University, Ankara, Turkiye; 2 Neuroscience and Neurotechnology Center of Excellence (NÖROM), Gazi University, Ankara, Turkiye; 3 Department of Physiology, Faculty of Medicine, Ankara University, Ankara, Turkiye; 4 Brain Research Center (AÜBAUM), Ankara University, Ankara, Turkiye; 5 Department of Neurology and Algology, Faculty of Medicine, Gazi University, Ankara, Turkiye; 6 Neuropsychiatry Center, Gazi University, Ankara, Turkiye; 7 Institute of Neuroscience and Neurotechnology, Gazi University, Ankara, Turkiye; 8 Department of Clinical Microbiology, Faculty of Medicine, Gazi University, Ankara, Turkiye

**Keywords:** Chronic migraine, Mig-SCog, inflammation, HMGB1, miR-146, IL-17

## Abstract

**Background/aim:**

Chronic migraine (CM) is a disabling neurological disorder associated with substantial impairment of quality of life. Increasing evidence suggests that inflammatory and epigenetic mechanisms may contribute to migraine pathophysiology. miR-146, a microRNA involved in the TLR-4/NFkb pathway, is known to regulate proinflammatory signaling, whereas HMGB1 activates inflammatory pathways through TLR2 and TLR4 receptors. IL-17 is another proinflammatory cytokine implicated in both inflammation and cognitive processes. In this research, our aim was to investigate the relationship between serum HMGB1, IL-17, and miR-146 levels and migraine-related subjective cognitive symptoms in patients with CM.

**Materials and methods:**

Serum samples were collected from 20 CM patients and 15 age-matched healthy controls. All the participants were female. Medical history, Mini-Mental State Examination (MMSE) scores, and migraine attacks - subjective cognitive impairments scale (Mig-SCog) scores were recorded. Serum HMGB1 and IL-17 levels were measured using an enzyme-linked immunosorbent assay. A quantitative real-time polymerase chain reaction was used to measure serum miR-146 levels. Statistical analysis of the data was conducted with SPSS 25.0. The relationships between Mig-SCog scores and serum HMGB1, IL-17, and miR-146 levels were investigated.

**Results:**

Serum HMGB1 levels were significantly elevated in the CM group compared to the control group (p = 0.012). However, no significant differences were observed between the two groups for serum IL-17 (p = 0.737) or miR-146 levels (p = 0.560). There was no significant correlation between HMGB1 and IL-17 (r = 0.167) or HMGB1 and miR146 levels (r = 0.073). Mig-SCog scores (p = 0.001) differed significantly between the groups and were positively correlated with HMGB1 levels (r = 0.403).

**Conclusion:**

Serum HMGB1 levels were elevated in CM patients and were associated with migraine-related subjective cognitive complaints, whereas no significant association was observed with miR-146 or IL-17 levels. These findings support a potential role of HMGB1 in migraine-related neuroinflammatory processes. Further studies are needed to clarify the relationship between inflammatory pathways and cognitive symptoms in migraine.

## 1. Introduction

Migraine is a neurological disorder affecting over one billion people worldwide per year [1]. It is considered chronic when headaches occur on more than 15 days per month; however, the diagnosis of chronic migraine (CM) still relies on clinical history, as no reliable biomarker has yet been established. On average, 3% of episodic migraine cases turn into CM annually [2].

Rather than being a static condition, migraine is dynamic with a fluctuating clinical course in terms of attack frequency, duration, and severity. In the pathogenesis of migraine, it is predicted that many genetic, epigenetic, and environmental factors have an impact [3]. Recently, epigenetic studies related to migraine pathogenesis and potential therapeutic targets have focused especially on DNA methylation, histone acetylation, and microRNAs (miRNAs) [4–6]. miRNAs are small endogenous RNAs involved in the regulation of gene expression and their role in migraine pathogenesis is an important issue at present [3].

Inflammation is another key area of interest for migraine researchers [7]. The role of inflammation and the innate immune system such as high mobility group box 1 (HMGB1) has been shown in CM [8–10]. HMGB1, which is a nuclear protein, functions as a damage-associated molecular pattern (DAMP) and is involved in the body’s alarm system against disturbances in homeostasis [11,12]. IL-17 is a proinflammatory cytokine that can be triggered by HMGB1 and is related to inflammation and cognition [13,14]. Various studies on cytokine levels in migraine patients have produced different results, but studies have generally shown that immunological changes may play a role in the pathophysiology of migraine [15]. We hypothesized that HMGB1, IL-17, and miR-146 expression may be associated with migraine-related subjective cognitive symptoms in CM.

Migraine patients frequently report symptoms such as confusion, difficulty finding words, mental slowing, difficulty concentrating, and memory problems during both ictal and interictal periods [16]. Although these symptoms may not always correspond to measurable impairment on formal neuropsychological testing, subjective cognitive complaints are increasingly recognized as an important component of migraine burden and may substantially affect daily functioning and quality of life.

In the present study, our aim was to investigate the relationship between serum HMGB1, miR-146, and IL-17 and whether these parameters are correlated with each other and Mig-SCog scores in CM patients.

## 2. Materials and methods

### 2.1. Study population and eligibility criteria

Female patients aged 18–65 years who fulfilled the International Classification of Headache Disorders, 3rd edition (ICHD-3) criteria for CM were consecutively recruited for the CM group. Age-matched healthy female volunteers without a history of primary or secondary headache disorders were included as controls. To reduce potential confounding effects, the participants in both groups were required to have no known neurological, inflammatory, or chronic systemic diseases. The exclusion criteria comprised additional neurological disorders; intracranial lesions or space-occupying pathology; secondary headache disorders; malignancy; severe metabolic syndrome; uncontrolled hypertension or diabetes mellitus; chronic liver, kidney, or respiratory disease; decompensated congestive heart failure; regular use of immunosuppressive therapy; alcohol or substance abuse; and major psychiatric disorders. All the participants provided written informed consent prior to enrollment. The study was conducted prospectively, and a priori power analysis indicated that 44 patients with CM and 44 healthy controls would be required. As the primary aim of the study involved circulating miRNA analysis, all serum samples initially underwent RNA isolation and quantitative real-time polymerase chain reaction (RT-qPCR) quality assessment procedures before molecular analyses were performed.

Despite repeated extraction and amplification attempts using different optimization approaches, sufficient RNA quantity and/or quality could not be obtained from all serum samples collected. Samples demonstrating inadequate RNA yield, absent or low-confidence amplification profiles, or technically inconsistent threshold cycle values were excluded according to predefined laboratory quality-control criteria.

Consequently, final molecular analyses were conducted using serum samples that met the analytical quality standards required for reliable miRNA quantification. The final study cohort consisted of serum samples obtained from 20 female patients with CM and 15 age-matched healthy female controls.

The study was conducted with 35 female participants who met the predefined inclusion criteria. Clinical characteristics and migraine-related cognitive symptoms were assessed using the migraine attacks - subjective cognitive impairments scale (Mig-SCog). Clinical migraine characteristics, including headache severity [visual analogue scale (VAS)], migraine duration, and monthly headache frequency, were also recorded and analyzed. Serum HMGB1 and IL-17 levels, as well as circulating miR-146 expression, were evaluated to investigate their association with cognitive symptom severity. The study protocol was approved by the Gazi University Clinical Research Ethics Committee (143-21.02.22) and was conducted in accordance with the Declaration of Helsinki.

### 2.2. Mig-SCog

Global cognitive status was additionally evaluated using the Mini-Mental State Examination (MMSE). The MMSE was administered to all participants as a brief screening tool for general cognitive function [17]. The Mig-SCog, developed by Gil-Gouvenia in 2011, is an accessible and reliable tool that assesses patients’ perceived cognitive decline during migraine attacks. This test uses a three-point Likert scale with scores ranging from 0 to 18. The first three questions focus on attention, processing speed, and orientation, while the fourth and fifth questions deal with planning and attention. Questions six to nine are related to language and naming skills [18].

### 2.3. Enzyme-linked immunosorbent assay (ELISA)

Samples were left at room temperature for 1 h before being centrifuged at 1000 × *g* for 20 min at 2–8 °C. Serum was stored at −80 °C after collection. High-sensitivity sandwich ELISA kits (Elabscience Biotechnology Inc., Houston, TX, USA) were used to measure serum HMGB1 (E-EL-H1554) and IL-17 (E-EL-H0105) levels. The intraassay and interassay coefficients of variation reported by the manufacturer were both <10%.

Standards and samples were added to micro-ELISA plate wells and combined and incubated with specific antibodies. Then biotinylated detection antibody, avidin-horseradish peroxidase conjugate, substrate solution, and stop solution were added consecutively in accordance with the kit protocol. The kit protocol was adhered to during the washing and incubation periods in the intermediate steps. By adding the stop solution, a yellow color reaction was obtained, and the microplate was read at 450 nm and optical density (OD) values were determined. A Combiwash Human ELISA plate washer was used for washing processes and OD values were determined with a ChroMate reader.

### 2.4. miR-146 expression

miRNA isolation was performed with a miRNeasy Serum/Plasma Kit (cat. no: 217184, QIAGEN, Hilden, Germany) from serum samples according to the protocol provided by the manufacturer. RNA samples were converted into complementary DNA (cDNA) by using a Mir-X miRNA First-Strand Synthesis kit (cat. no: 638313, Takara Bio, San Jose, CA, USA). A Lightcycler 480 II (Roche, Indianapolis, IN, USA) was used to perform RT-PCR with a Mir-X TB Green qRT-PCR kit (cat. no: 639676, Takara Bio, USA) and miRNA specific primer (TGAGAACTGAATTCCATGGGTT, Oligomer, Ankara, Türkiye). For normalization, U6 was used as the endogenous control, as it has been frequently used as an internal reference in RT-qPCR-based miRNA expression analyses, including circulating miRNA studies [19,20], and has also been used as an internal control in serum exosomal miRNA validation studies in migraine patients [21]. The forward and reverse primers of the U6 gene and universal reverse primer (mRQ) to amplify miR146 were included in the kit. The reaction conditions for PCR amplification were as follows: denaturation at 95 °C for 10 s and 40 cycles of amplification at 95 °C for 5 s and 60 °C for 30 s, followed by melting curve analysis. No-template negative controls were included in each run and each sample was run in duplicate. Relative miR146 expression was calculated by the 2-ΔCt method.

### 2.5. Statistical analysis

The results obtained from blood tests and the Mig-SCog scale were analyzed with SPSS Statistics 25.0 (IBM Corp., Armonk, NY, USA); p < 0.05 was considered statistically significant. Due to the relatively small sample size, the normality of data distribution was assessed using the Shapiro–Wilk test. Parametric data were analyzed with the independent samples t test and nonparametric data were analyzed with the Mann–Whitney U test. Then the relationships between the data were examined by creating a correlation matrix (Spearman correlation method). Tables were designed with GraphPad software. Parametric data were shown as mean ± SD, while nonparametric data were shown as median (Q1–Q3) values.

## 3. Results

### 3.1. Cognitive survey results

**Figure 1:**
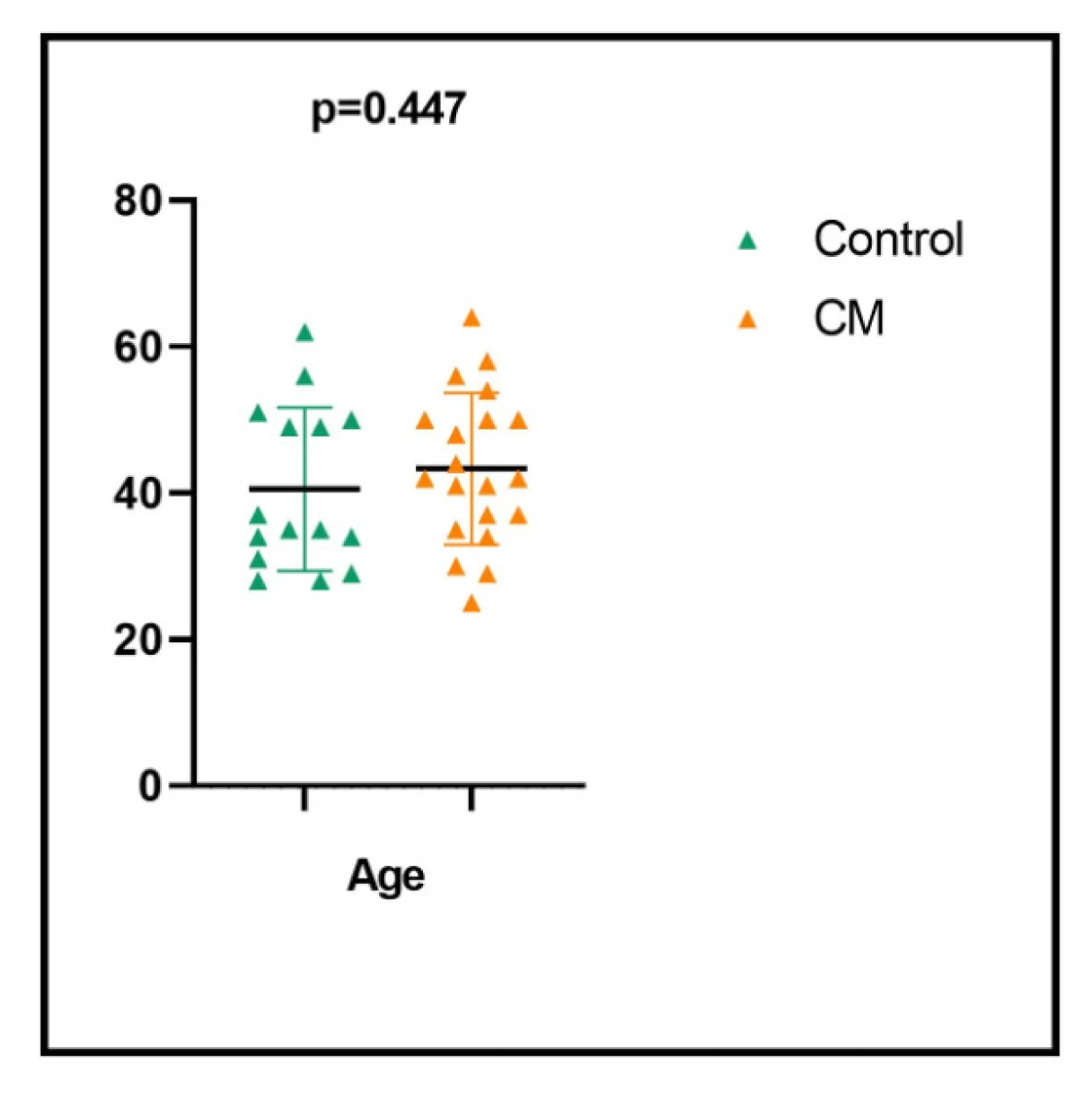
Age (years) was comparable between the CM patients and healthy controls (p = 0.447). Statistical analysis was performed using the independent samples t-test; p < 0.05 was considered statistically significant.

Twenty CM patients and 15 nonheadache healthy controls were included in the study. The mean age was similar between the groups (CM 43.35 ± 2.31 vs. control 40.53 ± 2.87, p = 0.447) (Figure 1). Mig-SCog scores were recorded in both the CM and control groups. Mig-SCog score (p = 0.001) was significantly higher in the CM patients. Clinical characteristics of the CM cohort were summarized descriptively. In the CM group, the median VAS score was 4 (2.25–5), median migraine duration was 300 (213–400) months, and median monthly headache frequency was 23 (20–30) days. Global cognitive screening scores were comparable between the groups. Mean MMSE score was 28.6 ± 1.2 in the CM group and 28.9 ± 0.9 in the control group.

### 3.2. ELISA results

**Figure 2:**
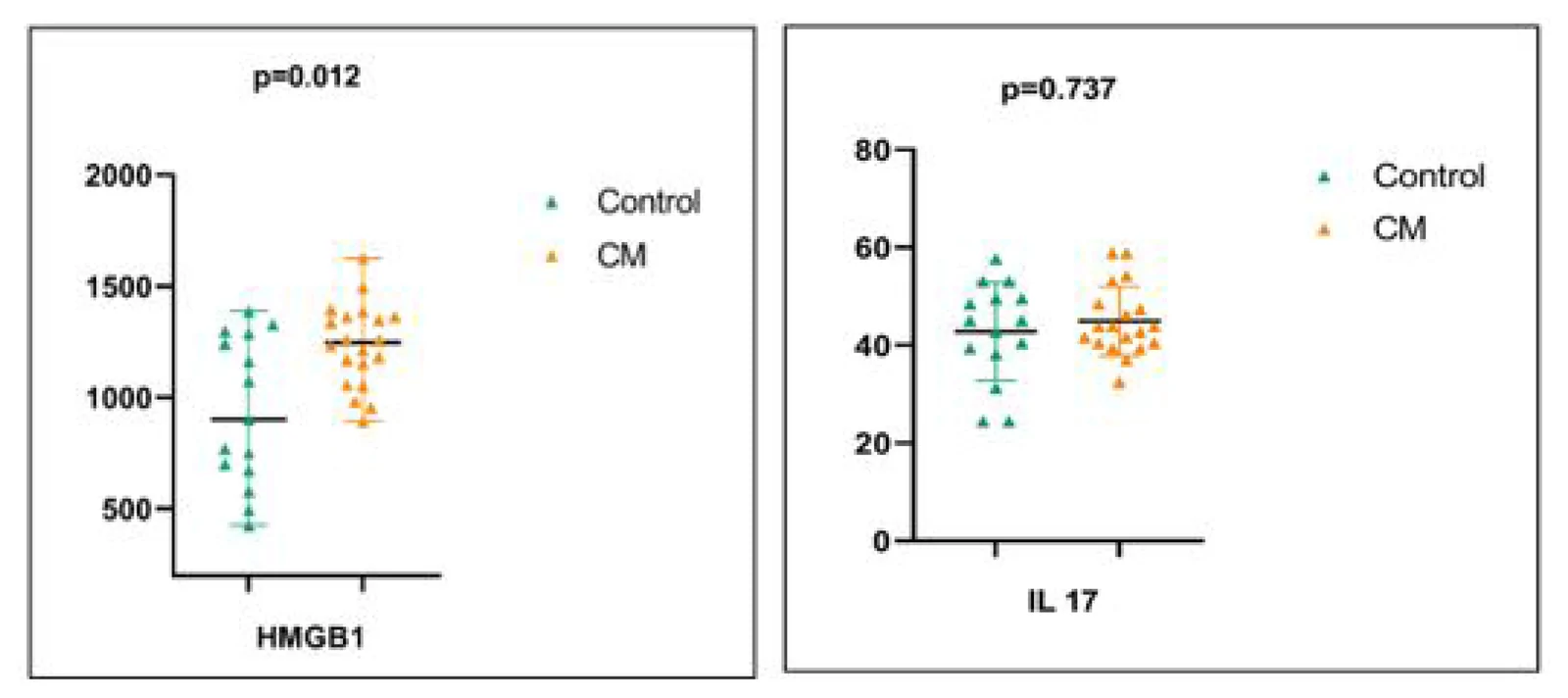
A) Serum HMGB1 (pg/mL) levels were significantly higher in the CM patients compared to the controls (p = 0.012). B) Serum IL-17 (pg/mL) levels were similar between the two groups (p = 0.737). Statistical analysis was performed with the Mann–Whitney U test for HMGB1 and the independent samples t-test for IL-17; p < 0.05 was considered statistically significant.

Serum HMGB1 and IL-17 levels were evaluated using ELISA and compared between the groups. Serum HMGB1 [CM 1257.10 (1055.72–1363.19) vs. control 988.11 (680.33–1274.85), p = 0.012] levels were significantly higher in the CM patients than the controls, while IL-17 (CM 44.948 ± 1.63 vs. control 43.928 ± 2.651, p = 0.737) were similar between the groups (Figure 2).

### 3.3. miR-146 results

**Figure 3:**
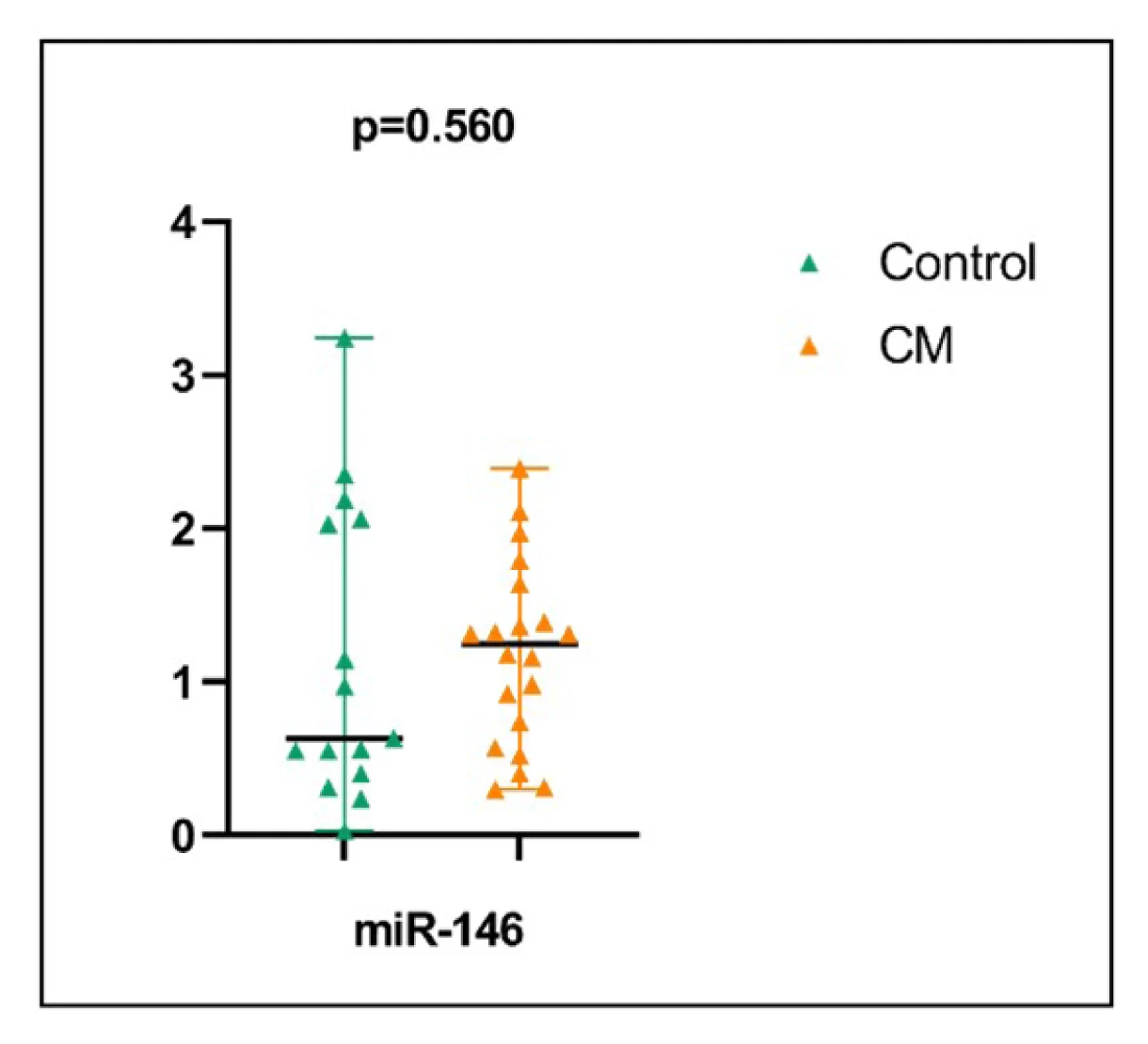
There was no significant difference between the two groups in terms of serum miR-146 levels (p = 0.560). Statistical analysis was performed with the Mann–Whitney U test; p < 0.05 was considered statistically significant.

Serum miR-146 levels were measured using RT-qPCR and no significant difference was observed between the two groups [CM 1.31 (0.57–1.64) vs. control 0.595 (0.3775–2.0925), p = 0.560] (Figure 3).

### 3.4. Correlation analysis

The relationships of all parameters with each other were determined by the correlation matrix. Diagnosis (1-control, 2-CM) was positively correlated with Mig-SCog score (r = 0.880, p = 0.0001). Serum HMGB1 levels showed a correlation with migraine-related cognitive burden (r = 0.403, p = 0.01), but not with serum levels of miR-146 (r = 0.073, p = 0.68) or IL-17 (r = 0.167, p = 0.33) (Table).

## 4. Discussion

CM is a highly prevalent and disabling neurological disorder worldwide, predominantly affecting women [1]. Despite ongoing research, the molecular mechanisms underlying CM remain elusive [22]. Epigenetic markers, especially miRNAs, are among the topics that attract attention for understanding migraine mechanisms [23]. miRNAs are regulators of fundamental processes in the nervous system and inflammatory systems, so they are likely to be involved in pain signaling as well [24]. Change in protein expression is one of the main features in the development of long-term hyperexcitability of peripheral nociceptive and central neurons, which may contribute to the development or persistence of chronic pain [25].

miRNAs are thought to have significant implications for inflammatory pain by influencing various processes, such as modulating glial cell activation and regulating inflammatory cytokines. miRNAs can be influenced by various environmental factors [26]. However, their precise role in migraine-related pain mechanisms remains incompletely understood, warranting further investigation [27].

miR-146 is a small RNA molecule that plays a role in many biological processes and controls the production of proinflammatory cytokines by regulating the nuclear factor kappa B (NF-κB) pathway [28,29]. NF-κB is essential for the regulation of acute phase protein transcription [30]. The relationship between migraine and inflammation has garnered significant attention in recent years, with numerous studies indicating an increase in inflammatory responses in serum, brain tissue, and the blood–brain barrier in migraine [8,9,31]. In this context, the levels of miRNAs, a key regulator of inflammatory processes, in migraine patients is a subject of considerable interest [32]. Studies suggest that miR-146 levels may exhibit variations in patients with migraine [33,34]. However, in the present study, no significant difference in miR-146 levels was observed between the groups, suggesting that its role in migraine pathophysiology may be influenced by factors such as patient heterogeneity, disease duration, or methodological differences in miRNA detection. miR-146 suppresses TLR4 while lipopolysaccharide (LPS) activates it [35]. In our previous studies, we demonstrated an increase in LPS levels in CM [8,9]. Additionally, HMGB1 exerts its effects by binding to TLR4 and activating the MAPK pathway. Disulfide HMGB1 activates the TLR4 complex by binding to myeloid differentiation factor-2 (MD-2), and this binding site is distinct from that of LPS [12,36]. In the present study, we examined serum miR-146 levels in migraine patients, a parameter that has not been extensively investigated. While we hypothesized a correlation between HMGB1 and miR-146, our findings did not support this association. This suggests that other molecular mechanisms or regulatory factors may be involved in this process, highlighting the need for further investigation. In this context, the potential effects of LPS and the complexity of this mechanism, which can be influenced by various molecules, should be taken into consideration. However, the effects of changes in miRNA levels on different migraine subtypes (e.g., migraine with and without aura) and their clinical implications are not yet fully understood [24].

HMGB1 is a nonhistone chromatin protein involved in numerous intracellular and extracellular biological processes [37]. Normally, it interacts with DNA in the nucleus to regulate gene expression [38]. However, under conditions of cellular stress or damage, it is released into the extracellular space, where it functions as a potent proinflammatory signaling molecule [39]. In this context, HMGB1 is recognized as a critical regulator that initiates and sustains inflammatory responses [12,40]. HMGB1 binding the toll-like receptors (TLRs) triggers mitogen activated kinase (MAPK) and thus creates its intracellular effects [12,41]. Activated MAPKs regulate intracellular enzymes, transcription factors, and cytosolic proteins that play a role in inflammation, the immune system, and apoptosis [41]. Various studies have shown an increase in inflammatory responses in brain tissue and its surroundings at the onset and during the duration of migraine attacks [10,42]. HMGB1, as a marker of inflammation, may play a potential role in the pathophysiology of migraine [8]. Other studies have shown elevated levels of HMGB1 in migraine [8,9,43]. In our study, serum HMGB1 levels were also higher in the patients with CM compared to the controls; this suggests that HMGB1 reflects the inflammatory component of migraine. Moreover, serum HMGB1 levels showed correlation with migraine-related subjective cognitive complaints. A better understanding of the role of HMGB1 in migraine pathophysiology may contribute to the development of new treatment approaches [44]. The relationship between migraine and serum HMGB1 levels may be an important step in understanding the inflammatory nature of migraine [45].

Cytokines are peptides that are the main mediators of inflammation [46]. IL-17 is a proinflammatory cytokine that can be triggered by HMGB1 and has an important role in immunity and inflammatory response [47,48]. It is thought that IL-17 may contribute to migraine pathophysiology by increasing inflammatory responses [31]. In addition, IL-17 has been proposed as a potential therapeutic target in various studies [49,50]. Therefore, our research included measuring serum IL-17 levels in patients with CM. However, no significant differences were found between the groups and IL-17 did not correlate with HMGB1 or miR-146. The lack of a significant association with IL-17 levels was possibly due to the small sample size. These findings suggest that the relationship between migraine and serum cytokine levels may be more complex than previously thought, possibly involving additional regulatory pathways.

One possible explanation for the isolated elevation of HMGB1 is that HMGB1 may represent a more upstream and relatively stable marker of neuroinflammatory activation in CM, whereas cytokines such as IL-17 and circulating miRNAs may exhibit greater temporal variability and may be influenced by attack timing, environmental factors, or technical preanalytical conditions. This may partly explain why HMGB1-related findings appeared more consistent in our cohort.

Importantly, the cognitive findings in the present study should be interpreted within the context of subjective cognitive symptoms rather than objective cognitive dysfunction, as Mig-SCog reflects patient-perceived cognitive difficulties associated with migraine.

Migraine-related subjective cognitive complaints were prominent among the patients with CM in the present study, as reflected by higher Mig-SCog scores, despite comparable global cognitive screening performance on the MMSE. Symptoms such as mental slowing, impaired concentration, attentional difficulties, and word-finding problems are frequently described by patients during both ictal and interictal periods. Although these complaints do not necessarily indicate objective cognitive impairment on formal neuropsychological testing, they may still have a meaningful impact on daily functioning and quality of life. In this regard, subjective cognitive symptoms appear to represent an important, yet often overlooked, aspect of migraine burden.

We also observed an association between higher HMGB1 levels and greater migraine-related subjective cognitive complaints. While the cross-sectional nature of the study does not allow causal inferences, this finding may support the idea that neuroinflammatory processes contribute not only to pain-related mechanisms but also to the cognitive symptom experience reported by patients with CM. At the same time, these findings should be interpreted with caution, since Mig-SCog reflects patients’ self-reported cognitive experiences rather than objective cognitive performance. Future studies combining inflammatory biomarkers with detailed neuropsychological assessment may help further clarify the relationship between neuroinflammation and cognitive symptoms in migraine.

Overall, our findings highlight the potential involvement of HMGB1 in CM pathophysiology, supporting its role as a key molecular player in neuroinflammation. However, the lack of association with miR-146 suggests that other regulatory mechanisms may be influencing this process. The lack of significant associations between HMGB1, IL-17, and miR-146 may indicate that these molecules reflect different aspects or temporal phases of neuroinflammatory activity in CM rather than a single linear biological pathway.

Recent studies further indicate that migraine is associated not only with pain but also with substantial cognitive and functional impairment affecting attention, memory, processing speed, and quality of life during both ictal and interictal periods. These findings emphasize the clinical relevance of investigating migraine-related cognitive symptoms and their potential biological correlates [51,52]. Therefore, evaluating inflammatory pathways associated with cognitive symptoms in CM may contribute to a better understanding of disease burden and symptom heterogeneity.

The relatively small sample size and the technical challenges specific to circulating serum miRNA analysis constitute limitations of the present study, as they prevented all samples collected from meeting the predefined quality control criteria necessary for reliable molecular evaluation.

Future studies with larger cohorts and mechanistic analyses are needed to further elucidate the complex interplay between inflammatory mediators and epigenetic regulators in migraine.

## 5. Conclusion

In this exploratory study, we demonstrated elevated serum HMGB1 levels in patients with CM, highlighting a potential role of HMGB1 in migraine-related neuroinflammatory processes. Notably, serum HMGB1 levels correlated with migraine-related subjective cognitive complaints but not with miR-146 or IL-17, suggesting that additional regulatory mechanisms may be involved in this process. These findings suggest that HMGB1 may be associated with neuroinflammatory mechanisms in CM and could represent a potentially relevant molecule for further investigation in larger prospective studies.

Given the complex and multifactorial nature of migraine, explaining its pathophysiology through a single molecular pathway would be insufficient. Therefore, further studies are needed to better clarify the interplay between inflammatory and epigenetic mechanisms in CM and to evaluate their potential clinical relevance.

## Figures and Tables

**Table:** Correlation matrix shows the interrelationship between serum HMGB1, IL-17, miR-146, and migraine-related disability. (Diagnostic: 1-Control, 2-Chronic migraine CM, High mobility group box 1 HMGB1, Interleukin 17 IL-17, migraine attacks - subjective cognitive impairments scale Mig-SCog, MicroRNA-146 miR-146, Visual analogue scale VAS, Migraine duration M.D., Headache frequency H.F.).

	Diagnostic	HMGB1	IL-17	MigSCog	miR-146	VAS	M.D.	H.F.
**Diagnostic**	1	0.432	−0.011	0.88	0.103	0.823	0.859	0.907
**HMGB1**	0.432	1	0.167	0.403	0.073	0.453	0.403	0.405
**IL-17**	−0.011	0.167	1	−0.008	0.079	0.071	0.228	0.128
**MigSCog**	0.88	0.403	−0.008	1	0.134	0.704	0.811	0.796
**miR-146**	0.103	0.073	0.079	0.134	1	0.107	0.09	0.176
**VAS**	0.823	0.453	0.071	0.704	0.107	1	0.75	0.838
**M.D**.	0.859	0.403	0.228	0.811	0.9	0.361	1	0.8291
**H.F**	0.97	0.405	0.128	0.796	0.176	0.838	0.829	1

## Data Availability

The data supporting the findings of this study are available from the corresponding author, Dr Hale Gök Dağıdır (halegok.dagidir@yuksekihtisas.edu.tr), upon request.
